# Empowering health volunteer’s through participatory action research in a comprehensive healthcare center

**DOI:** 10.1186/s12889-021-10878-7

**Published:** 2021-05-10

**Authors:** Fatemeh Vizeshfar, Marzieh Momennasab, Shahrzad Yektatalab, Mohamad Taghi Iman

**Affiliations:** 1 Department of Nursing, School of Nursing and Midwifery, Community Based Psychiatric Care Research Center, Shiraz University of Medical Sciences, P.O.Box:713451359, Shiraz, Iran; 2 Department of Nursing, School of Nursing and Midwifery, Shiraz University of Medical Sciences, Zand St, Namazi Sq., Shiraz, Postal Code: 7193613119 Iran; 3 Department of Sociology, College of Social Sciences, Shiraz University, Shiraz, Iran

**Keywords:** Empowerment, Health, Volunteers, Participatory action research

## Abstract

**Background:**

Health volunteers act as the link between the society and the healthcare system and are a symbol of people’s participation in the health maintenance and promotion. Despite the important role of health volunteers in the health system, it seems that they experience several problems. The aim of this study was empowering health volunteers through an action research.

**Methods:**

This participatory action research was conducted through two continuous cycles of reflection and acting for change over 20 months, in a comprehensive health center in Southern of Fars province in Iran. Participants included 25 health volunteers; two instructors and an academic researcher as facilitator. In the first cycle the participants discovered the challenges of health volunteers, prioritized them, designed an action plan, and implemented it. At the end of this cycle, evaluation was done with the participants’ cooperation. The second cycle began with reflecting on the results of the first cycle and then after designing and implementing second action plan, final evaluation was done.

**Results:**

Qualitative content analysis in the first phase led to the emergence of four categories, including role confusion, inadequate volunteer training, deficits in attracting and keeping volunteers, and being unfamiliar to the public. After the implementation of the action plan the participants learned through reflection and immersing in action. Finally, quantitative and qualitative data showed improvement of knowledge and performance, satisfaction, effectiveness of programs and improvement in volunteer’s competence.

**Conclusions:**

Clarification of volunteers’ roles, supporting volunteers to improve their knowledge and skills with coherent planning based on their educational needs, are appropriate management plans to attract and retain volunteers. By presenting their services to the society, they will be empowered and motivated to continue.

## Background

Health volunteers are people who spend their time and energy to serve other people and the society without receiving any money or financial reward [1, 2] . Many of them serve in healthcare settings [3]. Some countries use volunteers for implementing governmental programs to enhance public health [2–5]. Volunteers play an important role in improving people’s experience of care, create strong connections between the society and the services, facilitate care integrity, enhance public health, and reduce health inequality [6]. Many studies have been done on the positive effect of health volunteers’ performance and most of them show that they try to have an influential role in facilitating people’s access to personal, familial, and social health goals. Since health volunteers are role models in the society, they can have long term effects on people’s understanding, beliefs, and attitudes [7, 8].

One of the main problems of all organizations that use volunteers is continuous turnover due to their incoherent, irregular cooperation, and quitting [6, 9–11]. Several personal, social and health system factors affect the motivation and performance of health volunteers. Factors related to the health system include deficit in training and monitoring, insufficient financial rewards, high workload, insufficiency of services for the society, insufficient supervision, and insufficient support services such as transportation and job descriptions [1, 6, 10–13].

In Iran, health volunteers also face several problems that cause them to be separated from the health system. Lack of public familiarity with the volunteer program, lack of welfare and motivational facilities, and lack of attention to their suggestions and demands are among the volunteers’ challenges. On the other hand family support and opportunity for communicating to others through a social network are factors promoting volunteer cooperation [6, 7, 13, 14].

We aimed to identify the problems of health volunteers and empower them through a participatory action research. Action research is one of the methods of creating change which is used to facilitate and enhance service provision in the industry, education, and newly the healthcare systems. Action research is a research method that emphasizes on participation, participants, and empowerment in groups experiencing inequality. Other features of this method include being practical, transformational, and cyclic (through reflection). Thus, this method is suitable for research and change [15, 16].

### Health volunteers in Iran

In Iran, health volunteers are women selected and invited by local health care system from their residential area. They are literate and socially accepted, and have time, motivation, and interest for social activities. Each volunteer should cover about 50 families in their neighborhood. To gain necessary health-related skills and awareness, the volunteers participate in weekly meetings in their local health centers. They are trained by their instructor, who is a staff member of the comprehensive health center. Evaluation of the knowledge and performance of volunteers is also done by the instructor. The educational content was about child, maternal, familial, and social health and common diseases. Training consisted of primary and complementary courses. Health volunteers have an educational session every week. Besides these routine sessions based on their needs, smaller groups refer on other days to cooperate with the trainer and participate in screening programs, school training, family physician follow-ups, and other assigned duties.

One of the most important parts of the primary courses was identifying and prioritizing the neighborhood’s health issues. The complementary course starts after the volunteers begin their work and consists of various sessions and classes at the healthcare center. The course does not have any time limit. The volunteers and trainer agree upon the duration of the course and the time taken for each subject depends on the needs and conditions of the neighborhood. These sessions are provided with up-to-date knowledge about health-related issues. The subjects discussed are not limited and a wide range of issues are mentioned based on the volunteers’ needs and requests [8, 14].

## Methods

### Design

**Fig. 1:**
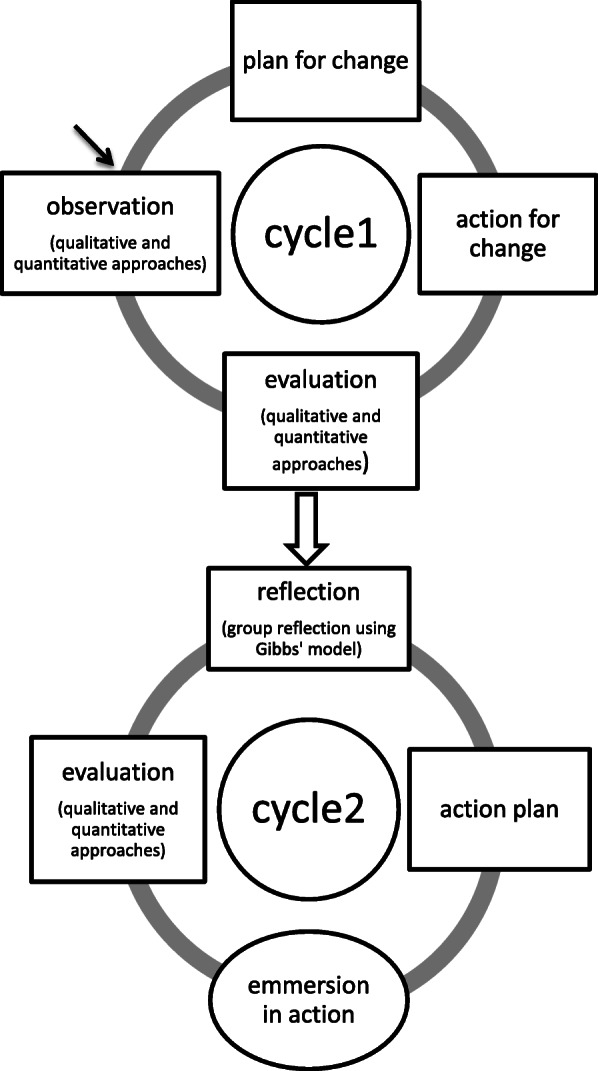
Cycles and steps of research process

This participatory action research was conducted in two cycles during 22 months from September 2016 to June 2018. Each cycle consisted of four stages (Fig. 1).

### Setting

This study was conducted in a comprehensive healthcare center in Shiraz, the capital of Fars province, southwest Iran. All primary and secondary prevention healthcare services are provided in this center. These services include child health, reproductive health and prenatal care, men and women’s health, adolescent health, elderly health, vaccination, neonatal screening, breastfeeding promotion, marriage counseling and thalassemia screening, mental health, nutrition counseling, environmental health, and occupational health. A family physician and a dentist also provide services to the population under coverage. The volunteers’ instructor not only teaches them, but is also responsible for school health and following and training family caregivers to the population under coverage.

### Participants

From thirty active volunteers in this setting, twenty-five who met inclusion criteria were selected by purposive sampling. The study inclusion criteria were having an active volunteer record in the Comprehensive Health Center and a history of more than 6 months as a volunteer. Moreover, two of volunteers’ instructors also participated. All participants were assured that refusing participation in this study, would not change their status as health volunteers. All the participants were informed about the study objectives and signed written informed consents for taking part in the study.

### Data collection

Since action research emphasizes on triangulation [15, 17–19], in this study, besides method triangulation, data triangulation was also used.

#### First cycle

##### Observation (first stage)

In the first stage of the first cycle, we determined the current working status of health volunteers including their strengths, limitations, and challenges, over 5 months. For this assessment qualitative and quantitative methods were used. Qualitative approach including recording and transcribing eight focused group sessions, eight in-depth interviews with the volunteers, two interviews with the instructors, 25 h of observation, and self-reporting journals. In quantitative approach 300 clients, who were came to this center for receiving health care services, were asked to complete a public survey tool which contain 6 questions regarding their information about and using volunteer services. Moreover, 30 health volunteers evaluated their own performance using a check list which is recommended by Health Vice-chancellor of Shiraz University of Medical Sciences.

##### Plan for change (second stage)

In this stage, according to the results of first stage an action plan for change was developed which took 3 months. In this stage the problems were determined and prioritized and an action plan was designed. Action plan was designed by participants and facilitator and approved by research team (Table 1).

**Table 1:** The designed action plan in two cycles

Problems	Goal	Intervention	Person	Evaluation
**First cycle**				
1- not having a clear plan	Organizing the health volunteer program	Volunteer Participation In Planning Designing course plan	Instructor Volunteers	Written course plan Volunteers’ satisfaction
2- disorganization in holding educational plans	Modifying the schedule of classes and programs	Designing an organized timetable for volunteers’ activities	Instructor Volunteers	Comparing the timetable with the implemented program
3- unclear job description	Providing job descriptions	Writing a clear and descriptive job descriptions	Instructor Managers Volunteers	Volunteers’ awareness about their duties
4- lack of practical skill training	Providing practical training program	Implementing theoretical and practical education about “first aid” and “checking vital signs”	Facilitator Researcher assistant Instructor	Observation using checklist Written test
5- deficit in training equipment and facilities	Improving access to resources	Renovating and completing hardware equipments Providing resources such as books and CDs	Instructor Managers	list of equipment
6- lack of public familiarity to volunteers program	Increasing public awareness	Volunteers introduce their work to people by media (pamphlets, etc.) More presence in public ceremonies such as “health week”	Volunteers	public survey
**Second cycle**				
1- lack of volunteers participation in education	More volunteers’ participation in their education	Providing a summary of previous and current lecture by the participants Expressing individual experiences for the class Encouraging learner for participation	Instructor Volunteers	Observation of classes
2- need for repeating practical training	Proper performance in first aid and checking vital sign	Repeating practical training for “first aid” and “checking vital signs”	Facilitator Researcher assistant Instructor	Observation using checklist Written test
3- deficit in volunteers’ familiarity with public healthcare services	Increasing volunteers’ awareness about primary and secondary healthcare services	Providing educational programs about primary and secondary healthcare services	Facilitator Instructor Volunteers	Oral exam
4- deficit in educational resources	Increasing volunteers’ ability for access to scientific resources	Providing education about search in electronic resources	Facilitator Volunteers	Homework assessment
5- deficit in communication skills	Promotion in communication skills	Holding an educational workshop on communication skills	Facilitator Instructor Volunteers	Volunteers’ satisfaction Observation using checklist

##### Action for change (third stage)

Immersion in action took 3 months. Health volunteers received training sessions and played their role with new knowledge and skill. The researcher and instructor encouraged and supported them. All volunteers cooperated with people and their instructor based on the plan to implement their duties such as screening and health education.

##### Evaluation (fourth stage)

At the end of the first cycle, the plan was evaluated using qualitative and quantitative methods. The progress in action plans and volunteer empowerment was determined through writing reflective journal, observing volunteer performance, interviews with volunteers, and recompletion of the 50 survey tool by people referring to the center.

#### Second cycle

This cycle took 6 months based on the results of the first cycle and its feedback. It consisted of reflection, action plan, immersion in action, and evaluation.

##### Reflection

This stage took 1 month and comprised four group reflection sessions. The initial evaluation results were shared with the volunteers. Gibbs model was used for reflection [20, 21].

##### Action plan

During 1 month, new goals for change were designed. Most of the aims included empowering the volunteers scientifically to facilitate their independence in learning based on the health system’s goals for this group (Table 1).

##### Immersion in action

During 3 months, the volunteers were immersed in action in which 12 sessions were held for them in the presence of their instructor and the researcher as well as higher level authorities and those referring to the healthcare center.

##### Evaluation

Ultimately, final evaluation was done. During this stage, the measures taken to empower the volunteers were assessed qualitatively and quantitatively. In the qualitative approach, six in-depth interviews and two focused group sessions were held for the volunteers. Their activities were observed twice and field notes were taken. Moreover, eight volunteers wrote reflective journal about their daily activities and the documents related to their performance were assessed. In the quantitative approach, 300 public survey tools were completed by the general public. Moreover, using Kirkpatrick’s model, the educational program of the volunteers was evaluated in four levels including their satisfaction with the program, knowledge gain, performance improvement, and effectiveness of the program. In the final level of the mentioned model, the effect of the educational program on the volunteers’ work environment was assessed according to their own view point by a questionnaire consist of 6 questions based on a 4-part Likert scale and its score was from a minimum of 6 to a maximum of 24.

### Data analysis

Quantitative data were analyzed using SPSS software, version 22, with descriptive statistics (mean, standard deviation) and inferential statistics (Paired t test for comparing mean score of learning). Conventional content analysis was used for qualitative data.

## Results

The majority of participating volunteer were married (80%) and housewives (88%). Their mean (SD) of age was 49.8 (8.65) years and mean (SD) of their work as a volunteer was 10.7 (4.0) years. Table 2 shows the demographic characteristics of the participants.

**Table 2:** The demographic characteristics of the participants

No.	Role	Education level	Job	Marital status	Husband’s job	Number of children	Years of volunteer work
P1	Volunteer	Middle school	Housewife	Married	Freelance	2	15
P2	Volunteer	Diploma	Housewife	Married	retired	4	5
P3	Volunteer	Primary school	Housewife	Widow	Freelance (dead)	4	15
P4	Volunteer	Primary school	Housewife	Married	Freelance	4	12
P5	Volunteer	Primary school	Housewife	Single	–	–	15
P6	Volunteer	College degree	Retired	Married	Accountant	3	8
P7	Volunteer	Middle school	Housewife	Married	Freelance	4	14
P8	Volunteer	Diploma	Housewife	Married	Teacher	2	15
P9	Volunteer	Bachelor’s degree	Housewife	Single	–	–	9
P10	Volunteer	Primary school	Housewife	Married	Freelance	4	14
P11	Volunteer	Diploma	Housewife	Married	Retired	4	15
P12	Volunteer	Middle school	Housewife	Married	Freelance	3	15
P13	Volunteer	Primary school	Housewife	Married	Freelance	2	12
P14	Volunteer	High school	Housewife	Married	Paramedic	3	3
P15	Volunteer	Middle school	Housewife	Single	–	–	5
P16	Volunteer	Middle school	Housewife	Married	Freelance	2	8
P17	Volunteer	Middle school	Carper weaver	Single	–	–	7
P18	Volunteer	College degree	Retired	Married	No job	5	12
P19	Volunteer	Diploma	Housewife	Married	Freelance	2	15
P20	Volunteer	High school	Housewife	Married	Freelance	4	15
P21	Volunteer	Primary school	Housewife	Widow	Freelance (dead)	3	15
P22	Volunteer	Middle school	Housewife	Married	Freelance	5	10
P23	Volunteer	Middle school	Housewife	Married	Retired	4	4
P24	Volunteer	Illiterate	Housewife	Widow	Freelance (dead)	4	10
P25	Volunteer	Diploma	Housewife	Married	Employee	1	12
P26	Current instructor	Bachelor’s degree	Healthcare worker	Married	Engineer	2	20
P27	Previous instructor	Bachelor’s degree	Expert	Married	Engineer	2	18

### Results of the first cycle

At the end stage of first cycle four categories emerged from qualitative data analysis including: role confusion, inadequate volunteer training, deficits in attracting and keeping volunteers, and being unfamiliar to the public. One main theme was also extracted named “unfertilized capacity”.

Results of the quantitative approach showed that in the first cycle of the study, the majority of volunteers (80%) were not properly aware of their own roles. Moreover, from volunteers’ viewpoint their main problems were as follows: not having a clear plan, disorganization in holding educational plans related to the volunteers, lack of practical skill training alongside theoretic instruction, unfamiliarity of the public with health volunteers, not benefiting from the volunteers’ abilities, and not having a place for holding the educational sessions. From the volunteers’ perspective most of the problems stemmed from lack of support from authorities and little interaction between the volunteer and their instructor. The results of public survey showed that 288 (96%) from 300 persons who were participated in the survey did not know that health volunteers even existed in the system and did not receive any services through them.

Participants designed and developed an action plan based on the determined and prioritizing problems. Table 1 shows the action plan. After implementing the plan, at the end of first cycle we found that the volunteers were eager to learn and perform their duties. Recompletion of the public survey tool showed that their unfamiliarity with health volunteers reduced from 96 to 88% and the volunteers put more effort into their work in this stage. Moreover, in the two interviews conducted, the volunteers emphasized on the need for the instructor’s support, and using participatory methods in education.

### Results of the second cycle

The second cycle of the action research began with the volunteers’ reflection. They expressed their experiences about the new knowledge and skills they had gained, the changes in their emotions and performance, the effects on their daily lives, and the lessons they had learned (Table 3). Also they reflected on the limitations of the first cycle and identified the problems and challenges they faced and designed a new action plan. A list of problems was prepared concerning issues such as lack of using participatory methods in education, deficits in health volunteer records and completing their performance check lists by the instructor, need for repeating practical training, lack of awareness about how to refer people to use healthcare facilities, deficit in their communication skills, and deficit in educational facilities.

**Table 3:** A participant reflection about the changes

During this time, as awareness grew, we were able to teach family, friends, and others the right training on many health issues. The right way we learned many things in these classes that we may not know how to do.	

After implementing the second action plan (Table 1), at the end of the second cycle we found that the public’s familiarity with health volunteers had increased from 4% (cyclec1) to 36% (cycle 2).

The results showed that most participants (*n* = 19, 76%) were satisfied with different aspects of the educational program. At the second and third level of Kirkpatrick’s model, the volunteers’ acquired knowledge and skill was compare using paired t test, indicating a significant increase in these domains (*P* = 0.0001). The scores ranged from 21 to 28 with a mean (SD) of 23.8 (1.92). We found that all volunteers perceived this course as effective. Comparing some items at the beginning of the first cycle and the end of second cycle revealed that significant personal and institutional improvement was happened during this participatory action research (Table 4).

**Table 4:** Comparing the changes at the beginning of the fist cycle and the end of the action research

Problems and Actions	Before the first cycle	End of the first cycle	End of the second cycle
Class order	Disorganized classes and constant cancellation without notice	Class cancellation without notice for two times	Orderly and organized class schedule, in case the instructor could not come (with prior notice) a substitute (an expert or another volunteer) would be introduced
Orderly presence of the volunteers in the classes	Disorganization, absence or on time presence, absence without prior notice	Less disorganization, most absence were without prior notice	Limited absence with prior notice
Interference in information communication and content transfer	Lack of order and access to information, being limited to contents of educational books	Creating groups in social media regarding the volunteers’ activities, using the group and educational pamphlets for transferring scientific content	Most volunteers had joined the group, they borrowed educational books and copied them, use of other valid sources
Location of the class	Home of volunteers	A room in the healthcare center	A room in the healthcare center
Content presentation in the class	Only by the instructor	Participation of both the instructor and the volunteer	Participation of both the instructor and the volunteer as well as other invited experts
Regular public training program	Very limited	Monthly sessions at the local center for religious activities, training at religious gatherings, recreational activities with family and friends	Qualitative and quantitative increase in public education, volunteers now have plans for public education themselves
Organized volunteer record keeping	Annually and only by the instructor	Every three months but still mostly by the instructor	Every three months with the guidance of the instructor
Volunteers’ sense of responsibility	Only for participating in the classes	Increased sense of responsibility for cooperating with the instructor and collecting family statistics	Increased sense of responsibility for cooperating with the instructor and collecting family statistics
Keeping track of issues with authorities	Limited and dispersed	Dome with more group participation and follow-up	Individual and group follow-up

At the end of the second cycle of the research, qualitative content analysis was performed on the transcripts of focus groups, in-depth interviews, reflective journals, and observations. Three hundred initial codes, 24 sub-subcategories, four subcategories, two categories, and one theme  were extracted for describing effects of the empowerment program on the volunteers (Table 5).

**Table 5:** The categories and subcategories of the program’s effects on empowering health volunteers

Theme	Category	Subcategory
Improving competence	Enhancing knowledge and skill	Effective instruction
		Skill improvement
	Controlled group performance	Facilitating communication and group work
		Support and mentorship

One of volunteer described her perceptions about the effect of this program as:

“*This program has excellent skills and everyone, even older volunteers with less literacy level is encouraged to participate. We did many things before, but it is more organized now*”. *(P16).*

Another participant said:

“*We know who the health volunteer is and what his responsibilities are. How can diseases be prevented? How to check clients’ blood pressure and blood sugar? It was so good. I wish it would continue”. (P23).*

Table 6 demonstrates the main points were mentioned in the focus group sessions by participants.

**Table 6:** The main points were mentioned by participants in the focus groups at the end of cycle 2

- The classes differed from before. We did not understand all the content before, but now we do.	
- It is a good experience to work together.	
-We must gain more information and skills.	
- The program was desirable and satisfactory.	
- There are still some problems in transferring health messages to people.	

## Discussion

The results of analyzing qualitative and quantitative data showed that the two-cycle program for empowering health volunteers increased their competence and independence. After the second cycle, health volunteers were empowered with respect to the skills required to perform their main tasks such as public education and cooperation in screening.

At the first stage of the study, qualitative data analysis led to the identification of four categories, which showed the problems of empowering volunteers including role confusion, inadequate volunteer training, deficits in attracting and keeping volunteers, and being unfamiliar to the public. These qualitative findings were supported by quantitative data.

One of the volunteers’ needs was better explanation of their roles and responsibilities and a coherent familiarity program. In some other parts of the world, the main challenges of health volunteers were that they were not fully familiar with their roles and were not supported for the services they provide [22]. It seems that increasing the volunteers’ awareness of their roles causes them to provide better services and help promote community health [23].

Moreover, in qualitative analysis, they mentioned that their educational programs were inadequate. Lack of practical skill training was a setback for empowering volunteers. In one study, the volunteers stated that there were several obstacles in providing volunteer care services for patients with syphilis, among which was lack of knowledge about services that should be provided [10]. The program planners must pay more attention to teaching the health volunteers’ required skills that could lead to their better performance [5].

Through qualitative analysis, we found that providing health services without basic and fundamental training as well as lack of educational facilities such as organized class schedule, necessary textbooks and equipment for teaching skills were the main problems volunteers faced. Providing necessary educational facilities increases their motivation and reduces the rate of quitting [24]. The healthcare system should plan for providing suitable training and improve their service providing standards [25]. The changes that had occurred at the end of the study were mostly related to volunteers’ educational needs and access to required theoretical and practical educational content. They found their instruction to be effective and their educational needs were met. Other researchers also found that education improved volunteer service providing in the healthcare system [5]. However, several studies in Iran and other countries showed that educational programs were not effective for health volunteers [10, 22]. The volunteers’ biggest challenge in our study was inadequacy of instruction. At the end of the study, we found that their knowledge had increased and they performed their duties much better than before. Volunteers’ satisfaction is an advantage of this program.

Inadequate support and supervision along with weak communication skills in volunteers and instructors were among other problems that the participants mentioned. In an ethnographic study in Uganda, the researchers found that the volunteers needed authorities to clarify how they should communicate with the healthcare team, local government, and medical structures [26]. Moreover, volunteers in south Africa stated that unsuitable communication with each other and inadequate team supervision were their weak points [27]. Lack of feedback on their activities was another issue the volunteers mentioned in our study. In another study in rural areas of Zambia, although volunteers had a positive view about their duties, their program had several weaknesses with respect to suitable supervision, positive feedback from the system, and facilities, which indicated that their program needed to be improved [28].

We found that the public was not familiar with the health volunteers. This leads to inadequate service providing since they are an important communication bridge between the health system and people requiring healthcare services. We think that Health volunteers perform their duties in the health system, but usually do not introduce themselves. While, Better familiarity with health volunteers increases people’s satisfaction and cooperation. For example, in China, most elderly welcomed volunteer care after being introduced to them and their programs [25].

In our study the volunteers were actively involved in needs assessment, which is the nature of action research. The results of a systematic review and meta-analysis on learning and participatory action in female volunteers for enhancing neonatal and maternal health in India, Bangladesh, Malave, and Nepal showed that their duties (which were based on participatory action and providing educational needs), reduced the rate of mortality in these regions [29].

Ultimately, data analysis at the end of the study yielded the main theme of “enhanced competence”. This is while the theme “unfertilized capacity” at the beginning of the study indicated lack of competence and capability which is consistent with many other studies [30–32]. We found that the volunteers’ capabilities had increased considerably leading to higher rates of effectiveness. This is in line with several other studies showing that volunteers have a high potential in promoting health-related and social welfare [30, 32–34].

### Limitations and future research

This study had several strengths such as method and data triangulation and using models for reflection and evaluation of program effectiveness. However, due to nature of action research as a context-based approach, the results cannot be generalized for other settings. So we suggest similar studies be done in other health centers in Iran and other countries. Future studies using the action research method on volunteers and other stakeholders would help identify and manage existing challenges in the health system.

## Conclusion

Clarification of the volunteers’ roles and supporting volunteers to improve their knowledge and skills with coherent planning based on their educational needs, are appropriate management plans to attract and retain volunteers. By presenting their services to the society, they will be empowered and motivated to continue.

## Data Availability

It is provided by request of the first author.

## References

[1] Taylor B, Mathers J, Atfield T, Parry J (2011). What are the challenges to the Big Society in maintaining lay involvement in health improvement, and how can they be met?. J Public Health.

[2] Jenner JR (1982). Participation,leadership,and role of volunteerism among selected women volunteers. J Volunt Action Res.

[3] Brudney JL (2000). Volunteers in State Government: Involvement, Management, and Benefits. Nonprofit Volunt Sect Q.

[4] Wing Keung J.L; Empowerment of non-academic personnel in higher education: exploring associations with perceived organizational support for innovation and organizational trust. 2010, University of Iowa. DOI : https://doi.org/10.17077/etd.nua1b3wl.

[5] Tulloch O, Taegtmeyer M, Ananworanich J, Chasombat S, Kosalaraksa P, Theobald S (2015). What can volunteer co-providers contribute to health systems? The role of people living with HIV in the Thai paediatric HIV programme. Soc Sci Med.

[6] Nylor C, Mundle C, Weaks L, Buck D; Volunteering in health and care securing a sustainable future,, ed. e. Rowling E. 2013, London: London King’s Fund.

[7] Volunteering in America, AmeriCorps,national s (2010). Corporation for National and Community Service.

[8] Shabaei F, Nik SN, Kshavarz N, Tohidi M (2016). Performance of health volunteers in Iran. J Islamic Repub Iran Med Organ.

[9] Scherer LL, Allen JA, Harp RE (2016). Grin and bear it: An examination of volunteers’ fit with their organization, burnout and spirituality. Burn Res.

[10] Bocoum FY, Kouanda S, Zarowsky C (2014). Barriers to antenatal syphilis screening in Burkina Faso. Pan Afr Med J.

[11] Hobfull SE (2011). Conservation of resource caravans and engaged settings. J Occup Organ Psychol.

[12] Farrer L, Marinetti C, Cavaco KY, Costongs C (2015). Advocacy for health equity: a synthesis review. Milbank Q.

[13] South J, Purcell ME, Branney P, Gamsu M, White J (2014). Rewarding altruism: addressing the issue of payments for volunteers in public health initiatives. Soc Sci Med.

[14] Alami A, Nedjat S, Majdzadeh R, Foroushani AR, Hoseini SJ, Malekafzali H (2013). Factors influencing women’s willingness to volunteer in the healthcare system: evidence from the Islamic Republic of Iran. East Mediterr Health J.

[15] Mcdonnell P, McNiff J (2016). Action research for nurses.

[16] Polit D. F, Beck CT, Nursing research generating and assessing evidence for nursing practice. 2017, Philadelphia.

[17] Koshy E, Koshy E, Waterman H (2011). Action researchin health care.

[18] Gilson L (2012). Health policy and systems research: a methodology ReaderAlliance for health policy and systems research. World Health Organ Action Res.

[19] Loewenson R, Flores W, Shukla A, Kagis M, Baba A, Ryklief A, Mbwili-Muleya C, Kakde D., Experiences of participatory action research in building people centred health systems and approaches to universal coverage, in Global symposium on Health Systems Research,; Montreux,Switzerland TARSC, Harare:. 2010, TARSC/ EQUINET, CEGSS SATHI-CEHAT.

[20] Asselin ME, Fain J (2013). Effect of reflective practice education on self-reflection, insight, and reflective thinking among experienced nurses: a pilot study. J Nurses Prof Dev.

[21] Tashiro J, Shimpuku Y, Naruse K, Mnwar M (2013). Miwako Matsutani., Concept analysis of reflection in nursing professional development. Jpn J. Nurs Sci.

[22] Gau YM, Usher K, Stewart L, Buettner P (2013). Burden experienced by community health volunteers in Taiwan: A qualitative study. Int J Nurs Pract.

[23] Kok MC, Kane SS, Tulloch O (2015). How does context influence performance of community health workers in low- and middle-income countries? Evidence from the literature. Health Res Policy Syst.

[24] Baker B.K., Benton D, Friedman E, Russell A., Systems support for task shifting to community health workers. Health Workforce Advocacy Initiative, 2007.

[25] Zhao L, Xie H (2015). Dong R; Volunteers as caregivers for elderly with chronic diseases: An assessment of demand and cause of demand. Int J Nurs Sci.

[26] Turinawe EBR, Rwemisisi TJ, de Groot M, Muhangi D, Mafigiri KD (2016). Towards Promotion of Community rewards to Volunteer Community Health Workers? Lessons from Experiences of Village Health Teams in Luwero, Uganda. Res Health Sci.

[27] Austin-Evelyn K, Rabkin M, Macheka T, Mutiti A, Mwansa-Kambafwile J, Dlamini T (2017). Community health worker perspectives on a new primary health care initiative in the Eastern Cape of South Africa. Plos One.

[28] Kambarami RA, Mbuya NM, Pelletier D, Fundira D, Tavengwa NV, Stoltzfus RJ (2016). Factors associated with community health worker performance differ by task in a multi-tasked setting in rural Zimbabwe. Glob Health Sci Pract.

[29] Prost A, Colbourn TM, Seward N, Azad K, Coomarasamy A, Copas A (2013). Women’s groups practising participatory learning and action to improve maternal and newborn health in low-resource settings: a systematic review and meta-analysis. Lancet.

[30] Schwarz D, Sharma R, Bashyal C, Schwarz R, Baruwal A, Karelas G (2014). Strengthening Nepal’s female community health volunteer network: a qualitative study of experiences at two years. BMC Health Serv Res.

[31] Singh D, Negin J, Garimoi Orach C, Cumming R (2016). Supportive supervision for volunteers to deliver reproductive health education: a cluster randomized trial. Reprod Health.

[32] Vareilles G, Negin J, Kane S (2015). Understanding the motivation and performance of community health volunteers involved in the delivery of health programmes in Kampala, Uganda: a realist evaluation protocol. BMJ Open.

[33] Gopalan SS, Mohanty S (2012). Das a; Assessing community health workers’ performance motivation: a mixed-methods approach on India’s Accredited Social Health Activists (ASHA) programme. BMJ Open.

[34] Srisarakhama P, Amnatsatsue K, Kerdmongkol P, Leerapanb P (2016). Development of a capacity building program for village health volunteers to support self-Management in a High Risk Population for diabetes in a rural Community in Northeast Thailand. Asian/Pac Island Nurs J.

